# *Aspidosperma nitidum* reduces parasite load and modulates cytokines in BALB/c mice infected with *Leishmania* (Leishmania) *amazonensis*

**DOI:** 10.3389/fchem.2024.1492770

**Published:** 2024-10-31

**Authors:** Heliton Patrick Cordovil Brígido, Everton Luiz Pompeu Varela, Antônio Rafael Quadros Gomes, Jorddy Neves Cruz, Juliana Correa-Barbosa, José Edson de Sousa Siqueira, Cristian Kallahan Silva Chagas, Andrey Moacir do Rosário Marinho, Liliane Almeida Carneiro, Márlia Regina Coelho-Ferreira, Sandro Percário, Maria Fâni Dolabela

**Affiliations:** ^1^ Postgraduate Pharmaceutical Innovation Program, Institute of Health Sciences, Federal University of Pará, Belém, Brazil; ^2^ Post-Graduate Program in Biodiversity and Biotechnology, Belém, Brazil; ^3^ Oxidative Stress Research Lab, Institute of Biological Sciences, Federal University of Pará, Belém, Brazil; ^4^ Center for Biological and Health Sciences, State University of Pará, Tucuruí, Brazil; ^5^ Institute of Biological Sciences, Federal University of Pará, Belém, Brazil; ^6^ Postgraduate Program in Chemistry, Institute of Exact and Natural Sciences, Federal University of Pará, Belém, Brazil; ^7^ Postgraduate Program in Pharmaceutical Ciences, Institute of Health Sciences, Federal University of Pará, Belém, Brazil; ^8^ National Primate Center, Ananindeua, Brazil; ^9^ Emílio Goeldi Museum of Pará, Belém, Brazil

**Keywords:** *Aspidosperma nitidum*, Apocynaceae, alkaloids, Leishmania amazonensis, Balb/c, IFN-γ, IL-10

## Abstract

The lack of vaccines shows the need for alternative leishmaniasis treatments. *In vitro* study previously demonstrated the leishmanicidal activity of *A. nitidum* extracts. This study describes for the first time, the antileishmanial activity of *A. nitidum* extracts in infected Balb/c mice and its immunomodulatory effect. The extract (EE) was obtained by maceration of the peel powder with ethanol, which was fractionated by acid-base partition, originating the alkaloid (FA) and neutral (FN) fractions. EE and FA were analyzed using mass spectroscopy. Daily intragastric treatment was performed with EE and FA, at doses of 200 mg/kg and 400 mg/kg, in Balb/c mice with 28 days of infection by *Leishmania amazonensis*. A thickness gauge was used to assess the progression of the lesion and the MTT method to determine the parasite load in the spleen. The quantification of IL-10 and IFN-γ was performed by ELISA. Analysis of the mass spectrum of EE indicated the presence of the alkaloids corynantheol and yohimbine, while in FA the alkaloid dihydrocorynantheol was identified. To elucidate the mode of interaction of these alkaloids with the TR protein, molecular target of antileishmanial drugs, we used molecular modeling approaches such as docking, molecular dynamics simulations and free energy affinity. Treatment with EE for 28 days at the highest dose tested, significantly reduced the size of the lesion. EE and FA after 28 days of treatment showed dose-dependent antileishmanial activity, which reduced the parasite load in the spleen of infected mice by 42.5% and 22.1%, respectively. Both EE and FA presented immunomodulatory effect, as they decreased IL-10 expression and increased IFN-y levels. The effectiveness of *A. nitidum* in the treatment of cutaneous leishmaniasis was proven in this study. The results obtained *in silico* demonstrated that the compounds are capable of interacting with the catalytic residues of the TR. The affinity energy results demonstrated that the complexes formed are favorable for enzymatic inhibition. The alkaloids present in the plant have demonstrated not only antileishmanial activity, but also the ability to modulate the host’s immune response. These promising results open perspectives for developing more effective and comprehensive treatments against cutaneous leishmaniasis.

## 1 Introduction

Cutaneous leishmaniasis (CL) is an infectious, non-contagious disease caused by protozoan parasites of the *Leishmania* genus. In clinical terms, CL is characterized by the formation of skin lesions that can spontaneously heal or evolve into a chronic condition, which can spread and lead to massive tissue damage, being commonly caused by species of *Leishmania braziliensis*, *Leishmania major,* and *Leishmania amazonensis* (Silveira et al., 2004; Goto and Lauletta Lindoso, 2012).

Leishmaniasis is endemic in 98 countries across tropical and subtropical regions, affecting approximately 350 million people at risk of infection. Each year, around 2 million individuals are infected, with cutaneous leishmaniasis (CL) responsible for an estimated incidence of 700,000 to 1 million new cases. The disease is linked to poverty, malnutrition, displacement, inadequate housing, immunosuppression, and lack of financial resources. CL presents a wide geographic distribution, with the majority of cases reported in Brazil, Afghanistan, Algeria, Colombia, Iran, Syria, Ethiopia, Sudan, Costa Rica, and Peru (World Health Organization, 2023).

Few drugs are available for the treatment of the disease. The first-line treatment for leishmaniasis are the pentavalent antimonials (meglumine antimoniate), the second line includes miltefosine, pentamidine, and amphotericin B (Tiuman et al., 2011). All antileishmanial drugs present several limitations, including severe side effects, the need for higher doses to achieve the therapeutical effect, high treatment costs and toxicity, with consequent low adherence to treatment and the emergence of resistance in strains of circulating parasites (Ghorbani and Farhoudi, 2018; Uliana et al., 2018). Due to these problems, many studies are still been carried out to find new alternatives. A promising line lies upon medicinal plants, and some studies have proposed new therapies for this disease.

*Aspidosperma nitidum*, is a tree that can reach up to 40 m in height, being found in the American continent extending in an area that goes from Panama to Brazil, it is popularly known as *carapanaúba* (Brazil), *jaroro hariraros*, *apokuita* and *padapan* (Suriname), and *gabetillo* (Bourdy et al., 2004). Species from the *Aspidosperma* genus are used by folk medicine in parasitic diseases such as malaria and leishmaniasis. They are also used in various infections and wounds that are difficult to heal (Bourdy et al., 2004; do Socorro Silva da Veiga et al., 2022).

To demonstrate the antileishmanial activity of *A. nitidum*, our research group has previously used the bark extract for phytochemical studies and *in vitro* assays against promastigotes and amastigotes of *Leishmania* species. The ethanolic extract (EE) and the alkaloid fraction (FA) were active against Leishmania chagasi promastigotes (IC_50_ < 100 μg/mL) and moderate activity against *Leishmania amazonensis* promastigotes (IC_50=_ 105–170 μg/mL; Veiga, 2013). Notwithstanding, against *L. amazonensis* amastigotes EE and FA) displayed high activity (IC_50_ = 23.87 ± 0.87 μg/mL and 18.5 ± 0.94 μg/mL, respectively). Moreover, the cytotoxicity assay in peritoneal macrophages from Balb/c mice revealed that both EE and FA presented moderate toxicity (CC_50_ = 491.8 ± 1.86 μg/mL and, respectively 209.1 + 1.7 μg/mL), but with promising selectivity (SI = 21 and 11, respectively) (Veiga et al., 2021).

In addition to *in vitro* studies, our group evaluated the *in vivo* toxicological potential of *A. nitidum*. The study of acute and subacute toxicity of EE and FA obtained from *A. nitidum* was carried out orally in Balb/c mice. The results demonstrated that both the single dose and the repeated doses treatments did not cause mortality or signs of toxicity in mice. In this context, the 50% Lethal Dose (LD_50_) of samples for mice was greater than 2.000 mg/kg in the acute test and greater than 1.000 mg/kg in the subacute test, suggesting a potential for safe use (Brígido et al., 2021).

Due to the limitations of current treatments for leishmaniasis, researchers are exploring plant-derived alkaloids as promising alternatives. Recent studies have highlighted the therapeutic potential of these compounds, demonstrating their efficacy in reducing parasite load and modulating immune responses (Sudarshan and Aidhen, 2013; Sudarshan et al., 2024). Thus, the ethanolic extract (EE) and alkaloid fraction (FA) from *A. nitidum* may serve as promising samples for efficacy studies in a murine experimental model with *Leishmania* infection, as they are selective and non-toxic for *in vivo* use. Based on these results, this study describes, for the first time, the antileishmanial activity of EE and FA obtained from *A. nitidum in vivo*, using Balb/c mice infected with *L. amazonensis*. Furthermore, it reports the immunomodulatory activity of these samples in infected animals.

## 2 Materials and methods

### 2.1 Plant material

Trunk bark of *A. nitidum* was collected on state highway PA-150 (coordinates S 02° 09′50.3″and W 048° 47′56.9″), in the state of Pará-Brazil, during August 2017. The plant material was identified by Dr. Márlia Regina Coelho-Ferreira and the exsiccate was deposited at the João Murça Pires Herbarium of the Museu Paraense Emílio Goeldi, under no. MG206608.

In the present study, we used a wild plant collected in an Amazon virgin forest, and our work did not represent an extinction risk for the species. During collection, we took great care to remove the bark without damaging the species, in addition, only a small part of the bark was collected to guarantee the tree survival.

Our study complied with national and international guidelines and legislation. It is registered on the platform of the National System of Management and Genetic Heritage and Associated Traditional Knowledge (*Sistema Nacional de Gestão e Patrimônio Genético e Conhecimentos Tradicionais Associados* - SISGEN), with license under registration A2C3188 for the collection of the species. Furthermore, according to the 2019 IUCN Red List of Endangered Species, *Aspidosperma excelsum*, a synonym of *A. nitidum* is classified as a “least-concern” species (Botanic Gardens Conservation International (BGCI) and IUCN SSC Global Tree Specialist Group, 2019).

### 2.2 Extract preparation and alkaloid fraction obtaining

The barks of *A. nitidum* were washed in running water and dried in a circulated air oven (40°C, for 7 days). The dried material was submitted to milling in a knife mill. The plant powder was submitted to maceration with 96°GL ethanol (ratio 1:10). The ethanolic solution was filtered and concentrated in a rotary evaporator under reduced pressure until total evaporation of the alcohol, obtaining the dry ethanolic extract (EE).

The EE was subjected to acid:base extraction in order to obtain the alkaloid fraction (FA). For this purpose, 5 g of the extract was solubilized in ethanol and subjected to partitioning, in a separating funnel, with an aqueous solution of 1N hydrochloric acid (HCl). This solution was extracted with dichloromethane (250 mL for 3 times), then the neutral fraction (FN) was obtained. The acidic aqueous layer was alkalinized with 10% ammonium hydroxide (NH_4_OH) to pH 9, followed by further extraction with dichloromethane (250 mL 3 times), obtaining an alkaline aqueous layer and an organic layer (FA), which was concentrated until residue.

### 2.3 Mass spectrum analysis

Mass spectra data were obtained by electrospray ionization (ESI) in positive ion mode using a Waters^®^ Acquity^®^ TQD instrument (Waters, Milford, MA, United States).

### 2.4 Animals

Seventy healthy adult male Balb/c mice (*Mus musculus*), aged between 6 and 8 weeks, weighing between 25 and 35 g, from the vivarium of Instituto Evandro Chagas (Ananindeua-Pará, Brazil) were used. The animals were housed in the Experimental Animal Facility of the Oxidative Stress Research Laboratory of the Institute of Biological Sciences of the Federal University of Pará (ICB/UFPA), on polypropylene cages (30 × 19 × 13 cm), covered with stainless steel grids, containing a pine bed, with a maximum of 5 animals per cage kept at room temperature (24°C ± 2°C) and light/dark cycle of 12 h each. Before and during the study period, the animals were kept with food (Presença, São Paulo-SP, Brazil) and water *ad libitum*. Before any experimental procedure, the animals were acclimated to laboratory conditions for 15 days. The experimental procedures with mice were carried out at the Oxidative Stress Research Laboratory (LAPEO/ICB/UFPA) in accordance with the ethical standards for animal experimentation indicated by the Brazilian Society of Laboratory Animal Science (SBCAL) and international standards (NRC National Research Council, 1996).

### 2.5 Ethics declaration

All animal procedures were strictly in accordance with the National Institutes Guide for the Care and Use of Laboratory Animals (NRC National Research Council, 1996) and approved by the Animal Use Ethics Committee of the Evandro Chagas Institute (CEUA-IEC), under the number 38/2017. Furthermore, this study was conducted according to ARRIVE guidelines (du Sert et al., 2020).

### 2.6 Parasites

The parasite used was *Leishmania* (L*.*) *amazonensis*, isolated from a human case from Ulianópolis, PA, brazil (MHOM/BR/2009/M26361) obtained from Instituto Evandro Chagas, Ananindeua-PA, Brazil. *L. amazonensis* promastigotes were obtained after primary isolation on the slopes of NNN (Novy-MacNeil-Nicolle) blood. The strains were then grown and adapted to Roswell Park Memorial Institute 1640 medium (RPMI-1640). The parasites were cultured at 26°C in RPMI 1640 medium supplemented with 10% heat-inactivated fetal bovine serum (Gibco^®^, Grand Island, NY, United States), penicillin (100 U/mL) and streptomycin (100 μg/ML) (Mota et al., 2015; Brígido et al., 2020).

### 2.7 *In vivo* infection of the murine model

BALB/c mice were infected with *L. amazonensis* promastigotes gathered in stationary phase (1 × 10^6^ cells in 50 µL PBS), intradermally injected in the right or left hind paw. The skin lesion measurement was performed from the 30th day after infection, and the size of the paw was evaluated weekly. For this purpose, a thickness gauge was used (Barão and Giorgio, 2003; Nakayama et al., 2005).

After infection, infected animals were randomly distributed into 7 groups (10 animals/group). Treatment started in the 8th week after infection and the arising of lesions. The extract and alkaloid fraction were administered in two water-diluted doses (200 mg/kg and 400 mg/kg), administered orally through an orogastric tube, once a day for 28 days. The negative control group was treated orally with water (sample vehicle; 1 mL/100 g). The positive control was treated with intraperitoneal injections of meglumine antimoniate (Glucantime^®^; 30 mg/kg/day) once a day for 28 days. The last group (control group) consisted of uninfected animals treated with vehicle (water 1 mL/100 g). Lesion sizes were measured twice a week using a caliper.

### 2.8 Parasite load quantification

It is noteworthy that *L.* (L.) *amazonensis* infection is not limited to the skin, the parasite tends to spread to lymph nodes and reach the spleen and liver (De Oliveira Cardoso et al., 2010). Therefore, we used the spleen, which was removed on the 14th or 28th days after the beginning of the treatment, for parasite quantification by limiting dilution and the animals were euthanized. The spleens were aseptically removed.

The organs were macerated and homogenized in RPMI 1860 medium with 10% fetal bovine serum (FBS). The cells were centrifuged at 1500 rpm, for 10 min, in a refrigerated centrifuge, and were plated at a concentration of 2 × 10^5^ cells/well in 96-well flat-bottom plates containing a final volume of 200 μL. Five serial dilutions (1:10) of the material were carried out. After 7 days incubated at 26°C, the plates were examined under an inverted microscope (Lima et al., 1997). The parasite load was quantified by the MTT method (3-(4,5-Dimethylthiazol-2-yl) 2,5 diphenyltetrazolium bromide), in which an aliquot of 100 μL of suspension from each well was homogenized with 10 μL of MTT (5 mg µg/mL diluted in PBS (Mosmann, 1983; Lima et al., 1997). The reaction was read at 490 nm (μQuant/Biotek Instruments INC. Winooski, United States).

### 2.9 Quantification of cytokines

The concentrations of the cytokines IL-10 and IFN-γ were evaluated in the animals’ plasma according to the study period. Cytokines were quantified using commercial kits by the ELISA method (Enzyme-Linked Immunosorbent Assay) and used according to the procedures previously described by the manufacturers.

Cytokine quantification was detected by colorimetry in a microplate reader at a wavelength of 492 nm, and the concentration for each sample was calculated from the corresponding standard curve in pg/mg of protein.

### 2.10 Histopathology

The skin of the animals’ paws was fixed in 10% buffered formalin and processed for inclusion in paraffin. Tissue sections (5 μm thick) were stained with Gomori’s trichrome (Almeida-Souza et al., 2016). The analysis was conducted on samples from all animals in each group to ensure comprehensive evaluation.

For the selection of the lesion area for analysis, a standardized approach was employed. The area of interest was specifically chosen based on the most prominent lesions, ensuring consistency across samples. Criteria for selection included lesion size, visibility of tissue changes, and proximity to unaffected skin, allowing for a comparative assessment of pathological changes.

Slides were evaluated by an independent certified histopathologist, and the results were confirmed by a second independent certified histopathologist. It is important to note that no statistical analysis was performed for this study, as the focus was primarily on qualitative histopathological evaluation.

### 2.11 Molecular docking

The molecular structures of yohimbine, corinanteol and dihydrocorinanteol were designed with the software GaussView 5.5 e optimized with Gaussian 09 (Frisch et al., 2024), using the Density Functional Theory (DFT) and B3LYP/6-31G* (Becke, 1993).

The molecular docking method was used to predict the binding mode of the molecules to the active site of Trypanothione reductase from *Leishmania infantum*. The docking was performed with the software Molegro Virtual Docker (MVD) 5.5 (Thomsen and Christensen, 2006), and the target crystal structure of Trypanothione reductase can be found in the Protein Data Bank with the following ID: 2JK6 (Baiocco et al., 2009).

The MolDock Score (GRID) scoring function was used with a grid resolution of 0.30 Å and radius of 10 Å, encompassing the entire connection cavity with its center at x, 17.85; y, 8.13; and z, −3.25. The MolDock SE algorithm was used with 50 runs, 3000 maximum interactions, and a maximum population size equal to 200. The maximum evaluation of 500 steps with a distance factor equal to 1 and an energy threshold of 100 was used in our protocol.

### 2.12 Molecular dynamics simulations

The molecular atomic charges were obtained with the Restrained Electrostatic Potential (RESP) protocol using the Hartree-Fock method with the 6-31G * base set (Cornell et al., 1993). The parameters for each molecule were constructed using the General Amber Force Field (GAFF) (Wang et al., 2004). Amber 16 package were used for the MD simulations (Case et al., 2005). The ff14SB force field (Maier et al., 2015) was used for all MD simulations. The absent hydrogens in the protein crystal were added by the tLEaP module during the process of building the complexes. The systems were solvated in an octahedron periodic box containing explicit water molecules described by the TIP3P model (Jorgensen et al., 1983). The distance chosen for the shear radius was 12 Å for all directions of the solvent from the solute.

The Particle Mesh Ewald method was used for the calculation of electrostatic interactions (Darden et al., 1993), and bonds involving hydrogen atoms were restricted with the SHAKE algorithm (Ryckaert et al., 1977). The simulation of MD was divided into stages of energy minimization, heating, equilibrium, and production. The sander module was used for both steps of energy minimization, where the steepest descent method and conjugate gradient algorithm were employed to perform 1500 cycles divided among the steps. In the first step, the solute was restricted with a constant harmonic force of 100 kcal/mol·Å-2, while the water and anti-ion molecules were free. In the second stage, the complexes were totally free to move.

Then, the systems were gradually heated for 600 ps until the temperature reached 300 K. The heating was divided into five stages, where the collision frequency was 3.0 ps^−1^ and the Langevin thermostat was used for temperature control (Lzaguirre et al., 2001). The heavy atoms were restricted with a constant harmonic force of 50 kcal/mol·Å −2 during the initial four steps. In the last heating step, the constant harmonic force was removed. These simulations were performed at constant volume (NVT). In the equilibrium stage, the systems were submitted to a simulation of 5 nanoseconds (ns) with a temperature of 300 K and constant pressure. During the production stage, 100 ns of MD simulations were generated.

### 2.13 Free energy calculations using the MM/GBSA approach

The free energy of each complex was obtained from the last 5 ns of the trajectory corresponding to 500 snapshots. In the MM-GBSA approach, binding free energy is calculated from the free energy of a linker interacting with a receptor to form the complex (Sun et al., 2014). Equation 1 is related to this phenomenon:

ΔG=ΔG ‐ ΔG – ΔG (1)

In each state, the free energy is calculated through the following expression, as shown in Equation 2:

ΔG=ΔG+ΔG ‐ TΔS (2)

ΔEMM is the energy of the total molecular mechanics in the gas phase, ΔGsolv is the free energy of solvation, and TΔS is the entropy of the system.

EMM represents the sum of the internal energy contributions (ΔEinternal, sum of the binding energies, angles and dihedrals), eletrostatic interactions (ΔEelectrostatic), and contributions of van der Waals (ΔEvdw), according to Equation 3:

ΔE=ΔE+ΔE+ΔE (3)

The free energy of solvation (ΔGsolv), as shown in Equation 4, is composed by polar (ΔGGB) and non-polar (ΔGSASA) contributions. Polar contributions are approximated by the Generalized Born (GB) method, and the non-polar contributions are determined from the calculation of the solvent-accessible surface area (SASA):

ΔG=ΔG+ΔG (4)

### 2.14 Statistical analysis

Statistical analysis was carried out using the Graph pad Prisma 5 program. For each parameter analyzed, an analysis of possible discrepant points (outliers) was carried out, using the interquartile range in its calculation, with discrepant points not being considered in the statistical calculations. For each parameter analyzed, the homoscedasticity of the dispersion was assessed, applying the Analysis of Variance (ANOVA) test for homoscedastic dispersion and the Mann-Whitney test when homoscedasticity was not met. After the existence of significant differences, they were compared between the groups, using Tukey’s *post hoc* test. In all tests, a significance level of 5% (*p* ≤ 0.05) was considered.

## 3 Results

### 3.1 Composition of *A. nitidum* extract and fraction

**FIGURE 1:**
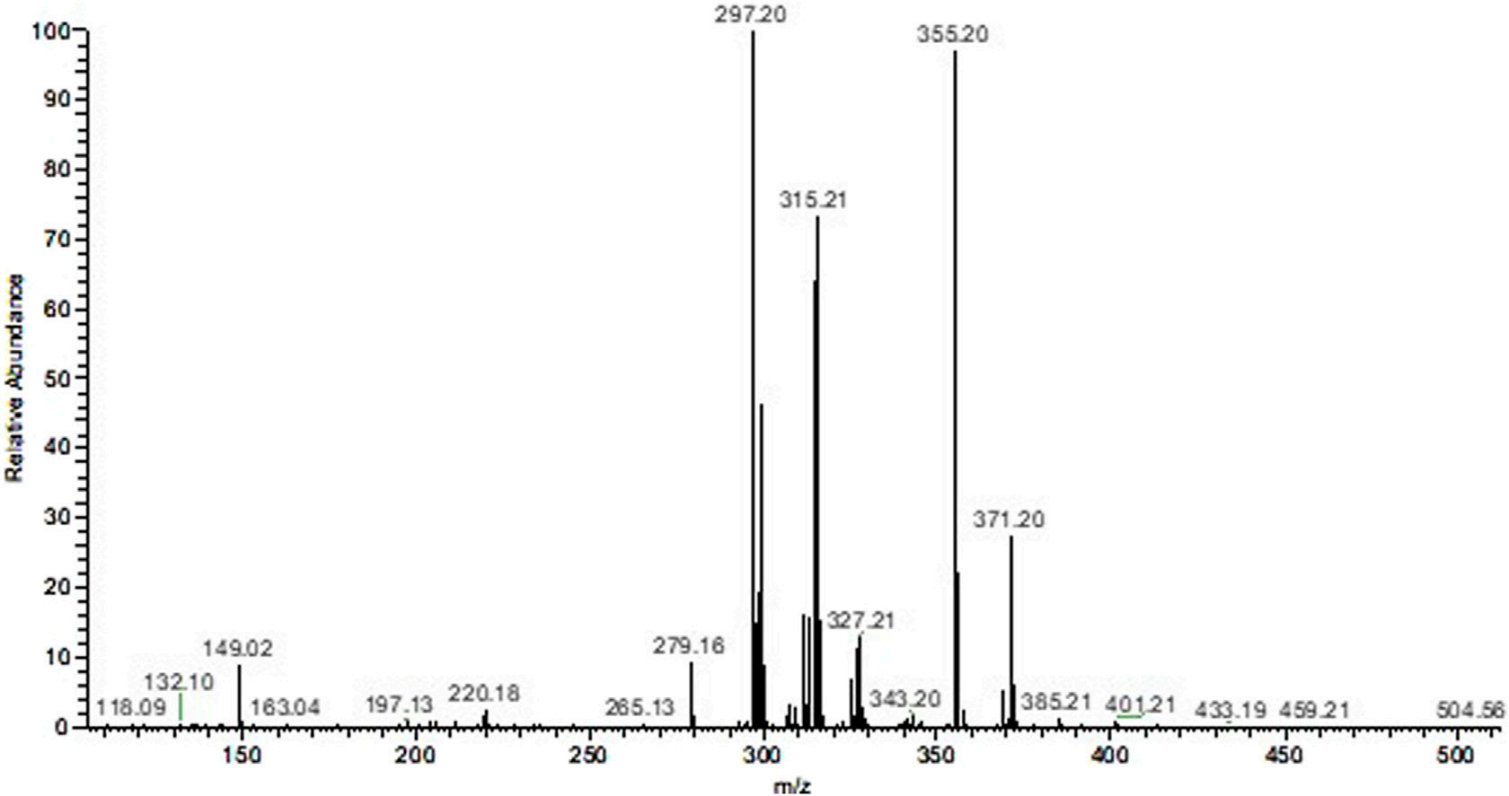
Mass spectrum of the ethanolic extract of *Aspidosperma nitidum*.

Scanning using the Mass Spectrometer of the ethanolic extract indicated the presence of two main constituents with m/z of 297.20 [M + H]^+^ and 355.20 [M + H]^+^. Based on these data and checking the literature it can be proposed that these are the alkaloids corynantheol (MM: 296.4 g/mol) and yohimbine (MM: 354.4 g/mol; Figure 1).

**FIGURE 2:**
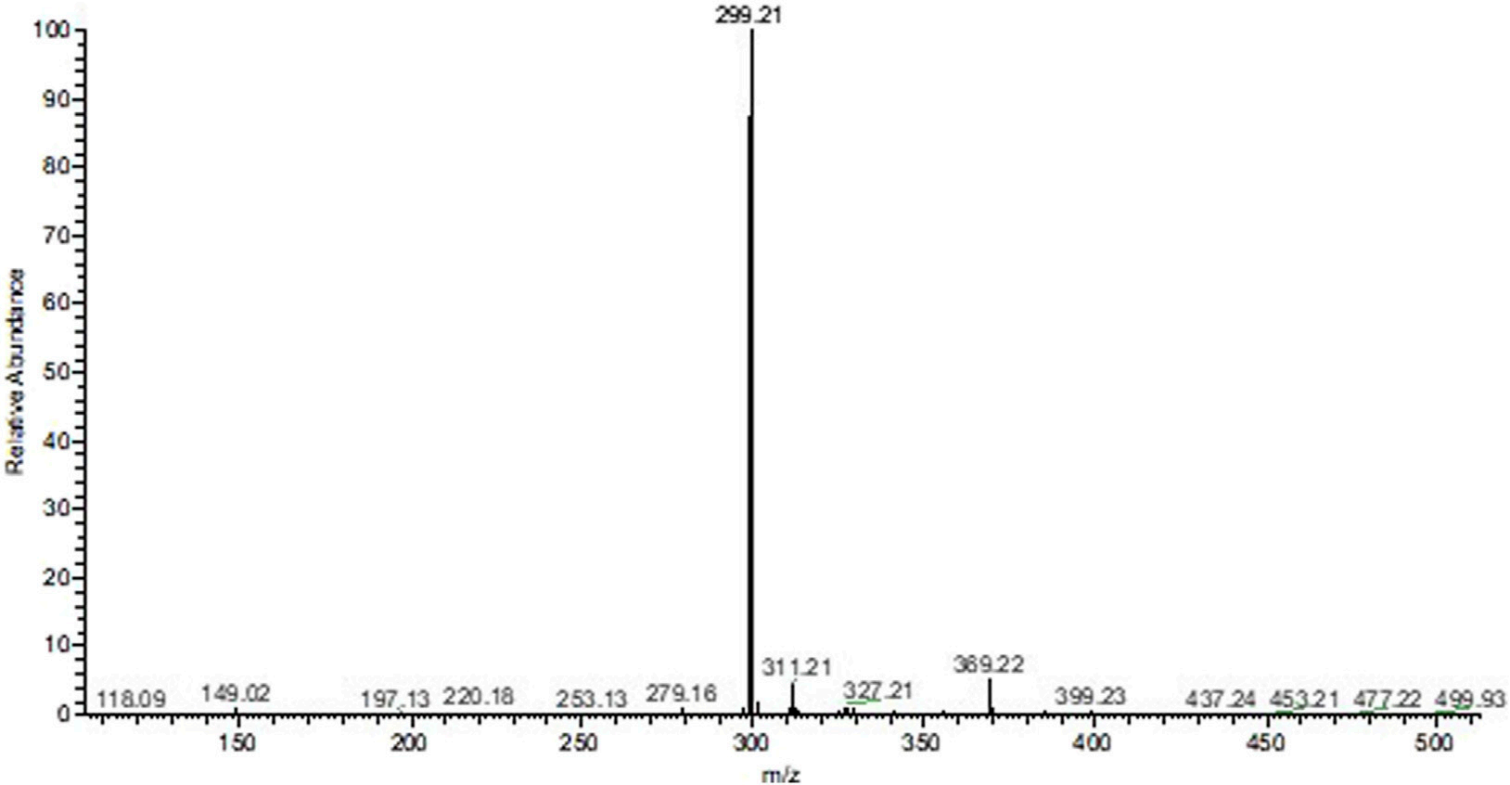
Mass spectrum of the alkaloid fraction of *Aspidosperma nitidum*.

As for the alkaloid fraction, a major constituent was identified with m/z equal to 299.21 [M + H]^+^, and based on the literature, it can be proposed that it is the alkaloid dihydrocorynantheol (MM: 298.43 g/mol; Figure 2).

### 3.2 Treatment with *A. nitidum* extract decreased lesion size

**FIGURE 3:**
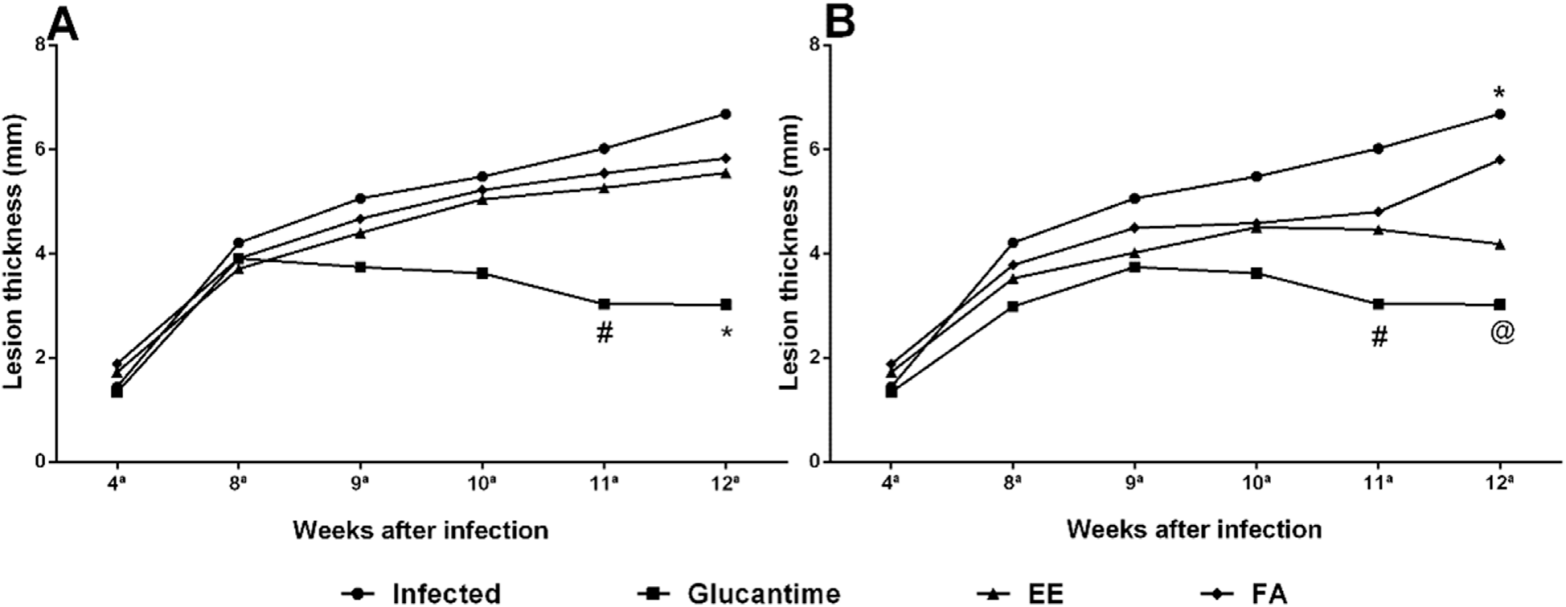
Evaluation of the cutaneous lesion in mice infected with *Leishmania amazonensis* treated with ethanolic extract (EE) and alkaloids fraction (FA) from *Aspidosperma nitidum*. Legend: **(A)** treatment with EE or FA 200 mg/kg; ^#^ *p* = 0.0213 versus Infected; *p = 0.0049 versus Infected and *p = 0.0313 versus FA; **(B)** treatment with EE or FA 400 mg/kg; #*p* = 0.0116 versus Infected and *p* = 0.0359 versus FA; *p = 0.0023 versus Glucantime and *p* = 0.0366 *versus* EE; ^@^ *p* = 0.0178 versus FA; EE: ethanolic extract; FA: alkaloids fraction; Glucantime^®^: 30 mg/kg/day intraperitoneally.

**FIGURE 4:**
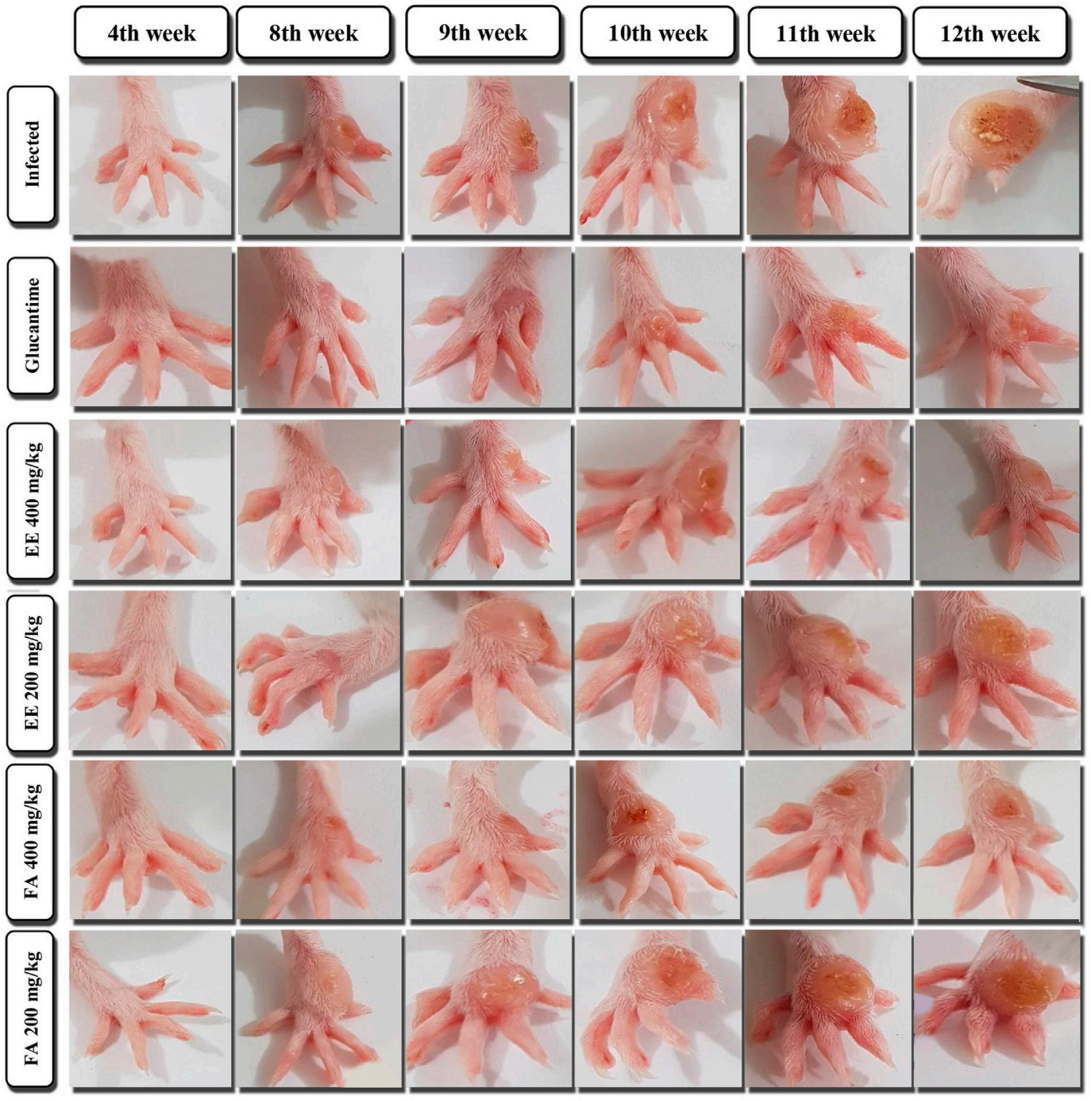
Evaluation of the cutaneous lesion of the paw in mice infected with *Leishmania amazonensis* treated with ethanolic extract (EE) and fraction of alkaloids (FA) obtained from *Aspidosperma nitidum*.

With the Glucantime^®^ treatment we observed a regression in the size of the paw lesion by 32.97% after 14 days (11 weeks) of treatment when compared to the control group. After 28 days (12 weeks) of treatment the decrease in lesion size became more pronounced, with 55.08% of regression (Figures 3, 4).

In the evaluation of ulcer healing in infected animals treated with EE, we observed the response was dose dependent. On the 28th day of treatment (12 weeks) there was a regression of 37.42% at the dose of 400 mg/kg. When mice received the dose of 200 mg/kg/day, a deceleration of lesion growth was observed (Figures 3, 4).

Unlike expected, FA did not reduce the size of the lesion induced by *L. amazonensis* when compared to the group of uninfected animals (Control group), however, there was a slowdown in ulcer growth (Figures 3, 4).

### 3.3 Treatment with the extract and alkaloids fraction from *A. nitidum* reduced the parasite load and presented an immunomodulating action

**FIGURE 5:**
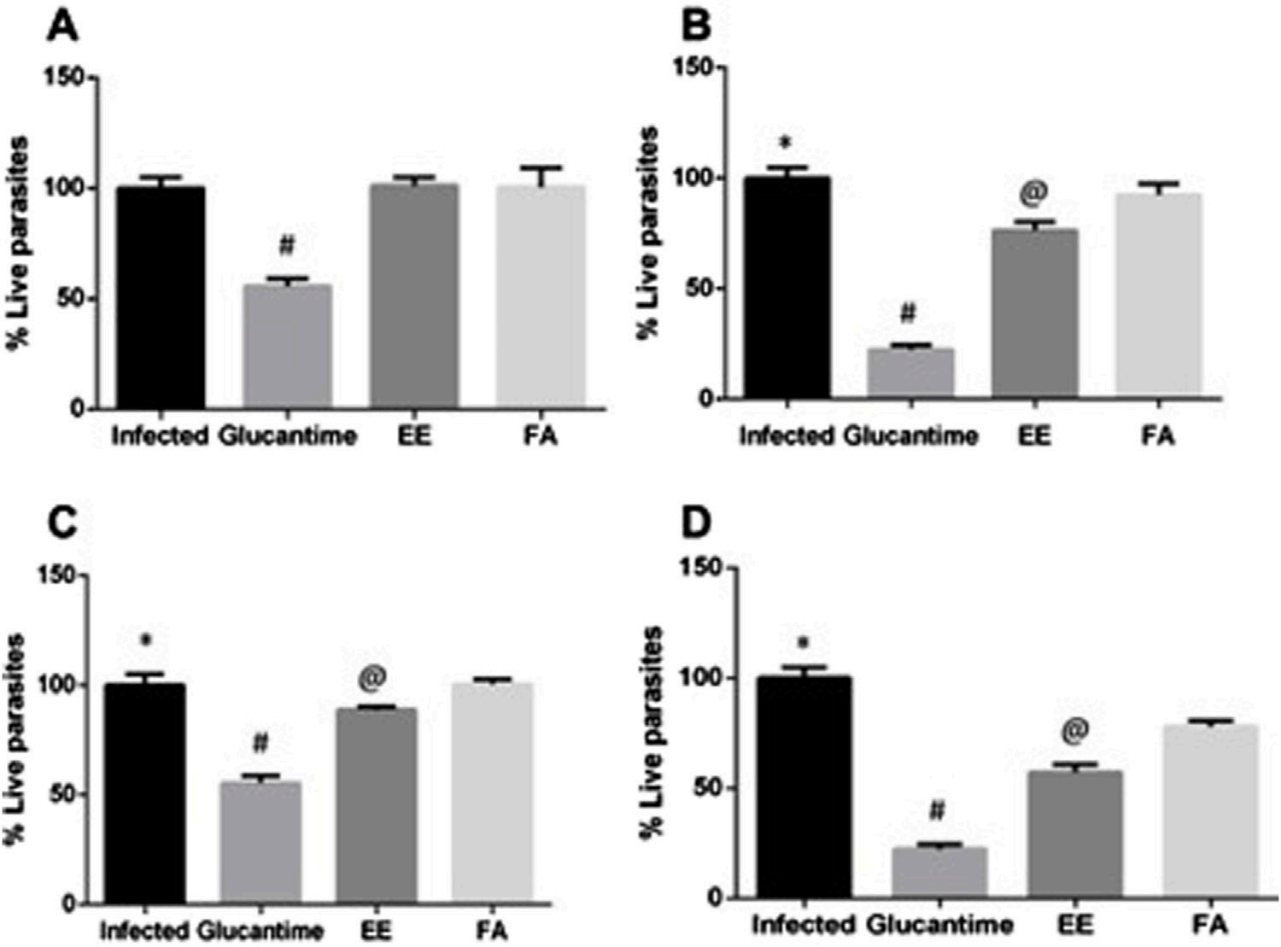
Parasite load in the spleen of mice infected with *Leishmania amazonensis* and treated with ethanolic extract (EE) or alkaloids fraction (FA) from *Aspidosperma nitidum*. Legend: **(A)** treatment with EE or FA 200 mg/kg for 14 days; #*p* < 0.0001 versus Infected, EE and FA; **(B)** treatment with EE or FA 200 mg/kg for 28 days; *p < 0.0001 versus Glucantime, EE and *p* = 0.005 versus FA; #*p* < 0.0001 versus EE and FA; @ *p* < 0.0001 versus FA. **(C)** treatment with 400 mg/kg for 14 days; *p < 0.0001 versus Glucantime and EE; #*p* < 0.0001 *versus* EE and FA; @ *p* < 0.0001 versus FA. **(D)** treatment with 400 mg/kg for 28 days *p < 0.0001 versus Glucantime, EE and FA; #*p* < 0.0001 versus EE and FA; @ *p* < 0.0001 versus FA. EE: ethanolic extract; FA: alkaloids fraction; Glucantime^®^: 30 mg/kg/day intraperitoneally.

For the EE, at a dose of 200 mg/kg/day, we observed a significant reduction (*p* < 0.0001) in parasitemia only after the 28th day of treatment (23.64%), when compared to the control group. At the dose of 400 mg/kg, we observed a significant reduction (*p* < 0.0001) of 11.4% in parasitemia on the 14th day of treatment and 42.56% on the 28th day. The reductions in parasitemia caused by EE were lower (*p* < 0.0001) than the reduction caused by Glucantime^®^ (Figure 5).

In animals treated with FA, we observed reductions only at the 28th day of treatment. When the infected animals were treated with 200 mg/kg/28 days, the parasitemia reduced by 7.69%. At the highest dose (400 mg/kg/28 days) a greater reduction was observed: 22.1% (Figure 5).

**FIGURE 6:**
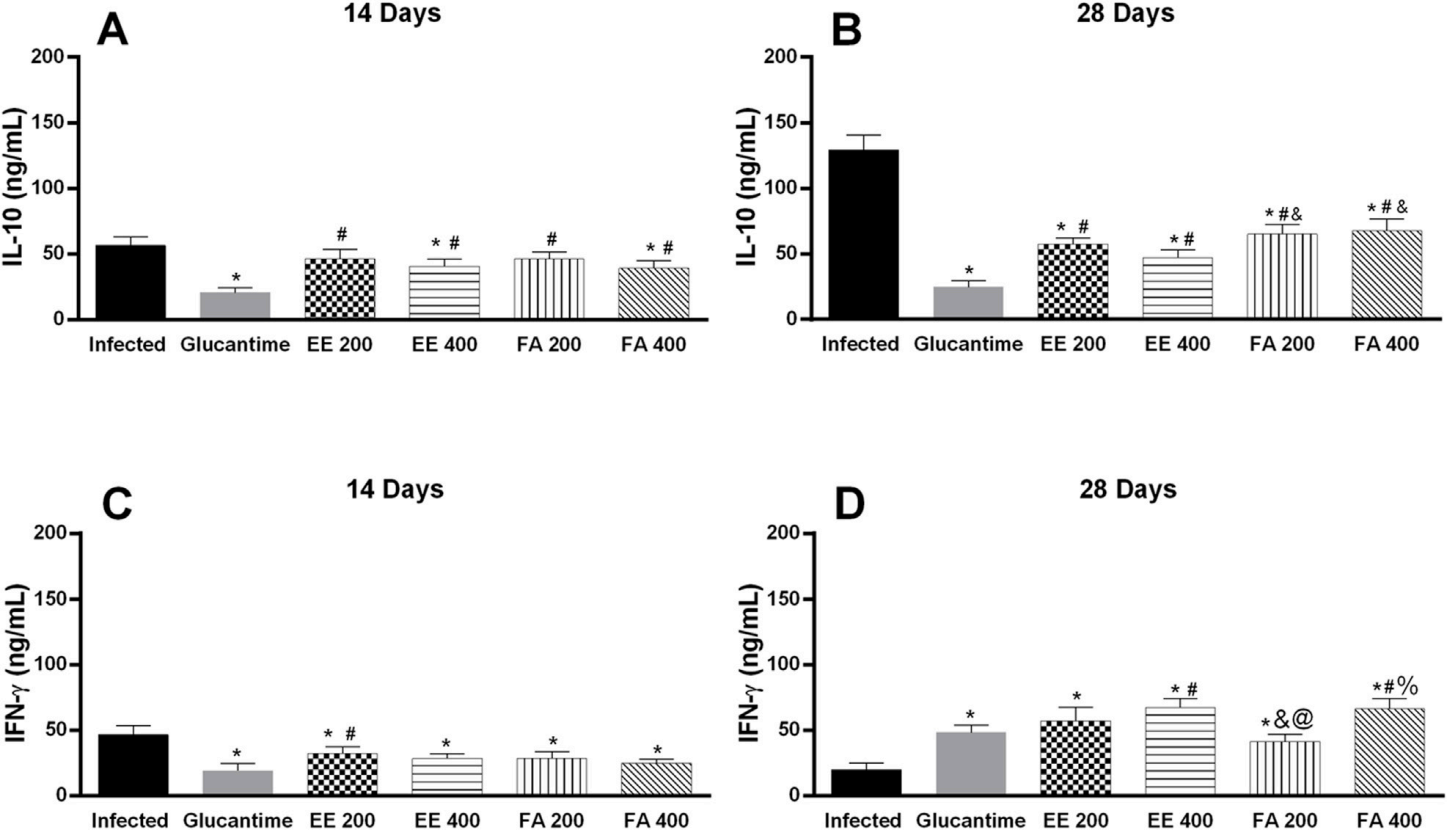
Quantification of cytokines in the serum of BALB/c mice infected with *Leishmania* (L.) *amazonensis* and treated with extract and alkaloids fraction from *Aspidosperma nitidum*. Legend: **(A)** IL-10 level in response to 14 days of treatment with 200 mg/kg; *p < 0.001 versus Infected; #*p* < 00,003 versus Glucantime. **(B)** IL-10 level in response to 28 days of treatment with 400 mg/kg; *p < 00,001 versus Infected; *#p* < 0.001 versus Glucantime; and *p* < 0,01 versus EE 400 mg/kg. **(C)** IFN-γ level in response to 14 days of treatment with 200 mg/kg; *p < 0.001 versus Infected; *#p =* 0.004 versus Glucantime. **(D)** IFN-γ level in response to 28 days of treatment with 400 mg/kg; *p < 0.001 versus Infected; *#p* < 0.004 versus Glucantime; and *p* < 0,02 versus EE 200 mg/kg; @ *p* = 00,001 versus EE 400 mg/kg; % *p* = 00,002 versus FA 200 mg/kg. EE: ethanolic extract; FA: alkaloids fraction. Glucantime^®^: 30 mg/kg/day intraperitoneally administered.

In the serum of infected and untreated animals (negative control) there was an increase in IL-10 levels, and on the 28th day we observed a more significant increase in the level of this cytokine. On the other hand, the parasite decreases IFN-y level according to the days of treatment. On the 28th day, IL-10 showed the lowest level in the infected and treated groups (Glucantime^®^, EE, and FA), and treatment with EE at a dose of 400 mg/kg, after Glucantime^®^, presented the smallest decrease in the expression of this cytokine. Finally, treatment with Glucantime^®^, EE, or FA increased the production of IFN-y after 28 days, and EE and FA at the dose of 400 mg/kg showed greater expression of the cytokine compared to Glucantime^®^ (Figure 6).

**FIGURE 7:**
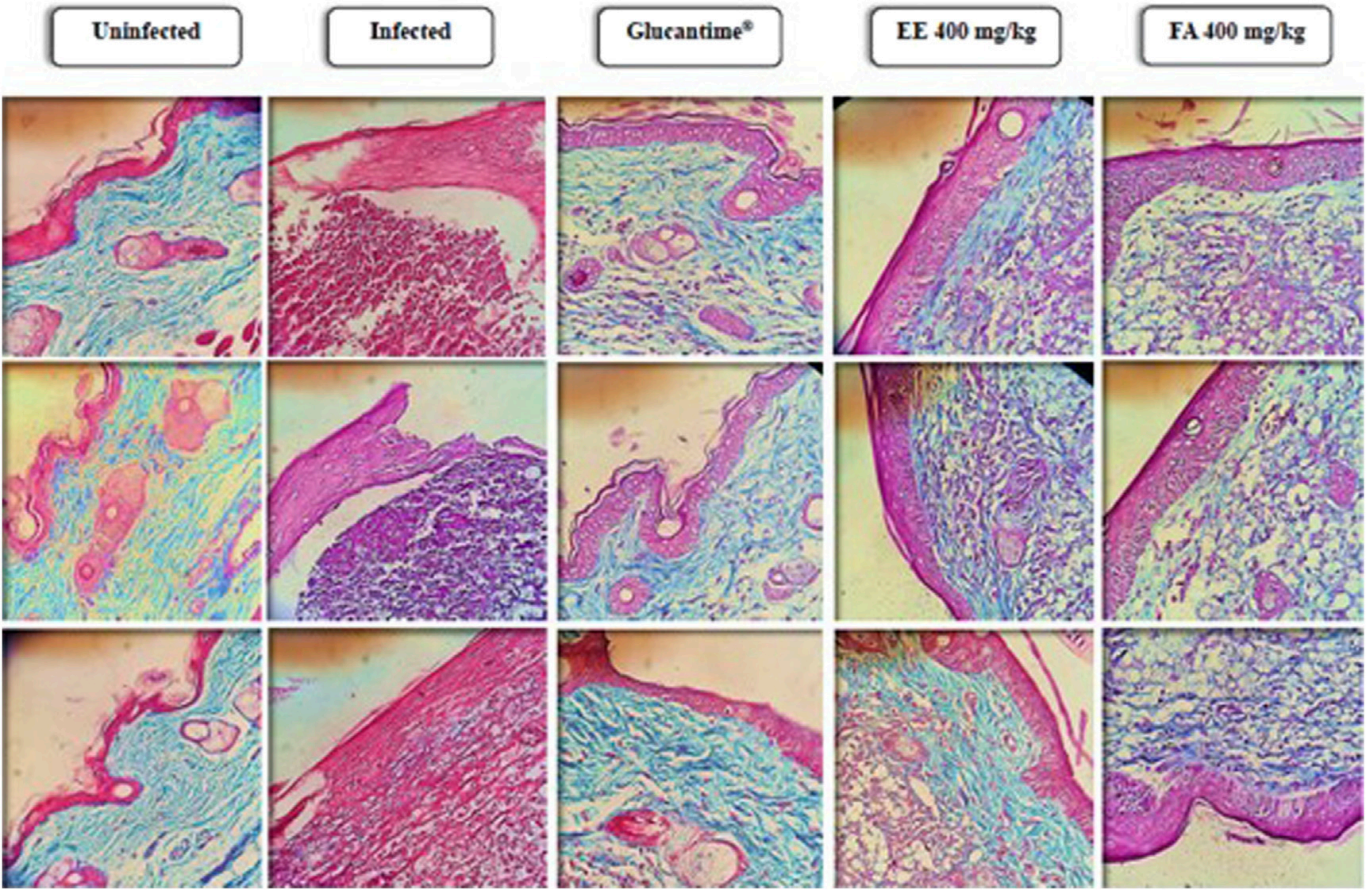
Histopathological analysis of the extracellular matrix of footpads from Balb/c mice infected with *Leishmania amazonensis* and treated for 28 days with ethanolic extract or alkaloid fraction obtained from *Aspidosperma nitidum* and matched controls. Legend: EE: ethanolic extract; FA: alkaloids fraction; Glucantime^®^: 30 mg/kg/day intraperitoneally administered.

The skin lesion site stained with Gomori’s Trichomium showed a reduction in the normal structure of the dermis and a degradation of connective tissue in the foot pads of infected animals without any treatment when compared to the group of uninfected animals. In addition, it was observed a reduction in the parasite load at the sites of injury in animals treated with Glucantime^®^, EE or FA, and an increase in collagen fibers when compared to the group of infected and untreated animals (Figure 7).

### 3.4 Molecular docking

To validate the docking methodology, we performed a redocking of the crystallographic ligand into the binding pocket of the Trypanothione reductase protein from the parasite *Leishmania amazonensis*. The fitness evaluation of the redocking was conducted using RMSD values and docking scores. The RMSD value between the crystallographic ligand and the redocked ligand was 1.3 Å. Docking experiments using known complexes with inhibitors that exhibit similar conformational complexity are typically performed to validate the docking protocol, following the recommendations of the Olson group. This process is crucial to ensure that the specified docking parameters in the input file are suitable and effective in accurately replicating the structure and interactions of a known complex (Forli et al., 2016; Silva et al., 2023).

The optimal conformation for the interaction of the compounds yohimbine, corinanteol, and dihydrocorinanteol was evaluated based on the Moldock score results. The most favorable conformation for each compound yielded Moldock scores of −65.37, −76.69, and −74.08, respectively (Table 1). The conformations obtained from molecular docking studies demonstrate that these molecules were able to interact with the catalytic residues of the molecular target, such as Cys52, Cys57, His461’, and Glu466’. These residues are essential for the redox mechanism of the protein (Baiocco et al., 2009; Battista et al., 2020).

**TABLE 1:** Moldock scores obtained from the docking protocol using MVD 5.5.

Molecule	Moldock score
Yohimbine	−65.37
Corinanteol	−76.69
Dihydrocorinanteol	−74.08

### 3.5 Molecular dynamics (MD) simulations

The complexes obtained from molecular docking were used as the initial coordinates for the molecular dynamics (MD) simulations. All complexes underwent energy minimization, heating, system equilibration, and production MD protocols, resulting in 100 ns trajectories.

**FIGURE 8:**
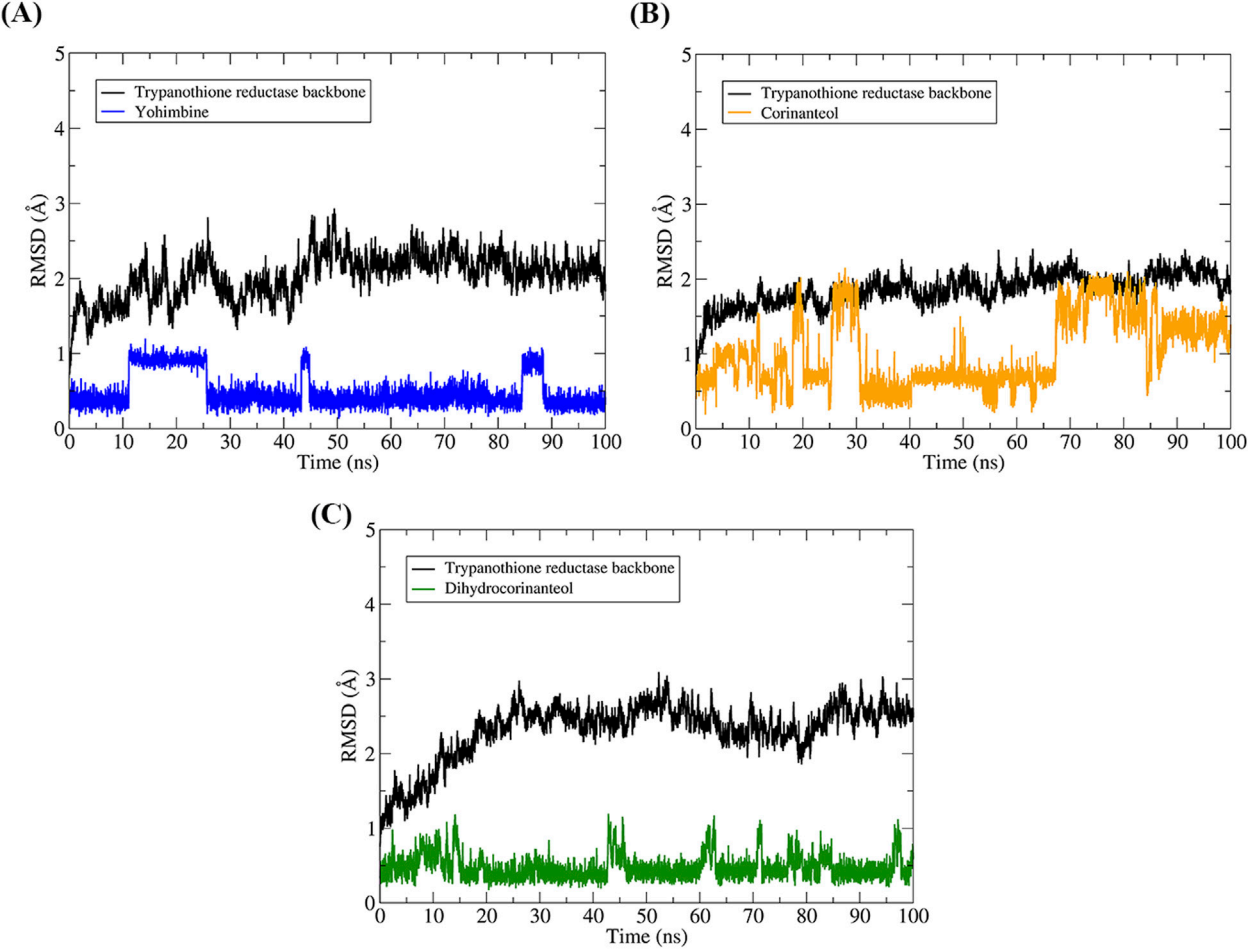
Analysis of the conformational stability of the systems over 100 ns of MD simulation. The protein backbone is represented in black in all graphs, while the colors representing the binders vary. The RMSD graphs were plotted in relation to the systems obtained after the steps of minimization, heating, and equilibrium. TR-yohimbine in blue **(A)**, TR-corinanteol in yellow **(B)**, TR-dihydrocorinanteol in green **(C)**.

The structural stability of the complexes was assessed using the RMSD values of the protein backbone and the ligands (Figure 8). The RMSD for yohimbine showed that the ligand experienced structural modifications between 0.5 and 1 Å, indicating minimal conformational changes throughout the simulation trajectory. The RMSD for corinanteol demonstrated structural shifts between 0.5 and 1 Å, followed by an increase to 1–1.5 Å. However, in the last 30 ns, the ligand maintained a stable conformation with only minor structural modifications. The RMSD for dihydrocorinanteol remained around 0.5 Å throughout the trajectory, with no significant conformational changes. As illustrated in Figure 8, all ligands remained within the binding pocket of the molecular target during the entire 100 ns of MD simulations and continued to interact with the catalytic residues.

### 3.6 Affinity energy of complexes

We used the MM-GBSA approach to evaluate the interaction energy of the three complexes. MD simulations of 100 ns generate many conformational configurations, so we selected the simulation interval where the RMSD showed the most stability. Therefore, for these calculations, we used the last 10 ns of MD simulations for each complex.

The results are summarized in Table 2. According to the data, the interaction of yohimbine, corinanteol, and dihydrocorinanteol with the TR protein was favorable, with binding affinity energies of −21.72, −20.21, and −22.15 kcal/mol, respectively. The contributions from van der Waals interactions (ΔE_vdW_), electrostatic energy (ΔE_ele_), and nonpolar solvation energy (ΔG_NP_) were favorable for the stability of the TR-ligand complexes. Among these contributions, van der Waals interactions were the most significant contributors to complex formation, with values of −26.85, −18.45, and −27.90 kcal/mol for yohimbine, corinanteol, and dihydrocorinanteol, respectively. Nonpolar contributions (ΔG_NP_) were also favorable, though their impact was minimal.

**TABLE 2:** Affinity energy values (values in kcal/mol). ΔE_vdW_, contributions by van der Waals interactions; ΔE_ele_, electrostatic energy; ΔG_GB_, polar solvation energy; ΔG_NP_, nonpolar solvation energy; ΔG_bind_, binding affinity.

Molecules	ΔEvdW	ΔEele	ΔGGB	ΔGNP	ΔGbinding
Yohimbine	−26.85	−4.42	13.14	−3.59	−21.72
Corinanteol	−18.45	−9.87	10.32	−2.21	−20.21
Dihydrocorinanteol	−27.90	−12.57	21.71	−3.38	−22.15

## 4 Discussion

Phytochemical studies carried out on different parts of *A. nitidum* revealed the presence of several alkaloids. Among them, 10-methoxy-dihydro-corynantheol, corynantheol (Arndt et al., 1967), aspidospermine, quebrachamine, yohimbine (Marques et al., 1996), harman carboxylic acid, 3-carboxylic ethylharman (Pereira et al., 2007), dihydrocorynantheol, dehydrositsiriquine and braznitidumine (Brandão et al., 2010). In a previous study carried out by our research group, both EE and FA obtained from *A. nitidum* were subjected to analysis by high-performance liquid chromatography (HPLC) (Brígido et al., 2021). In the EE chromatogram, indole alkaloids and β-carboline were identified, corroborating previous findings (Arndt et al., 1967; Bolzani et al., 1987). Indole alkaloids, with varying structure, are frequently isolated, many of which have a simple β-carboline skeleton (Wenkert, 1962; Allen and Holmstedt, 1980). On the other hand, in the FA chromatogram, only peaks suggestive of indole alkaloids with an aspidospermine nucleus were observed (Pereira et al., 2007). It is worth mentioning that aspidospermine has been associated with antiplasmodial, antileishmanial and antitrypanosomal activities (Coatti et al., 2016).

Analysis of the mass spectrum of EE indicated the presence of the alkaloids corynantheol and yohimbine, while in FA the alkaloid dihydrocorynantheol was identified. Such compounds, such as yohimbine, corynantheol and dihydrocorynantheol, have already been identified in previous studies with *A. nitidum* (Arndt et al., 1967; De Oliveira Cardoso et al., 2010; do Socorro Silva da Veiga et al., 2022).

Indole alkaloids are recognized for their antileishmanial activity. Previous studies demonstrated that alkaloid fractions rich in this class did not present acute toxicity at a dose of 2,000 mg/kg, nor subacute toxicity at a dose of 200 mg/kg for 28 days (Brígido et al., 2021). Furthermore, some of these compounds, such as dihydrocorynantheol, have been shown to cause changes in the flagellar pouch and cytoskeleton of *Leishmania*, leading to the death of the parasite and a consequent reduction in parasitemia (Veiga et al., 2021).

After the infection of Balb/c mice with *L. amazonensis*, the different experimental groups received the treatments from the 8th week, because the appearance of a small skin lesion in all groups inoculated with the parasites was only visible after this timeframe. Initially, the lesion was characterized by edema and a small erythema, with slow, but progressive growth. In fact, when treatment started, all lesions were about 2 mm thick.

The Glucantime^®^ treatment significantly reduced lesion growth from the second week of treatment onwards, with lesion regression becoming more pronounced in the fourth week. In the treatment of human beings with american tegumentary leishmaniasis, the effect of this drug is also time dependent, requiring prolonged treatment (Moreira et al., 2017), which contributes to the emergence of adverse reactions and toxic events (Oliveira et al., 2011).

When seeking therapeutic alternatives for the treatment of leishmaniasis, it is expected to obtain drugs that promote ulcer healing faster than current use antimonials with less toxic potential. This therapeutic alternative also should be orally administered to enable high administration and to reduce the final treatment cost.

The ulcer healing and reduction of the parasite load of animals treated with EE from *A. nitidum* was dose and time dependent with better effect on the 28th. On the other hand, FA did not reduce the size of the lesion induced by *L. amazonensis*, however, there was a slowdown in ulcer growth, but there was a slight reduction in the parasite load on the 28th day.

When analyzing the chemical compositions of *A. nitidum*, previous phytochemical studies using HPLC-DAD indicated the likely presence of β-carboline and indole alkaloids in the extract, while an alkaloid fraction would be composed mainly of indole alkaloids (Brígido et al., 2021). In the present study, we identified the alkaloids corynatheol and yohimbine in the EE, while in the FA we found the alkaloid dihydrocorynatheol. Therefore, the antileishmanial activity of *A. nitidum* seems to be more related to these alkaloids (Brígido et al., 2020). An issue that needs to be better understood is the role of the immune response in this process, as a previous study demonstrated the *in vitro* antileishmanial activity of the β-carboline alkaloid flavopereirine with the results of molecular docking showed flavopereirin was able to inhibit oligopeptidase B (da Silva e Silva et al., 2019).

IFN-γ plays a crucial role in the control of *Leishmania* infection, the cytokine induces parasite clearance by activating the phagocyte oxidase (phox) and iNOS enzyme complex, which is the most effective mechanism to kill intracellular parasites mediated by macrophages (Murray and Nathan, 1999; Salaiza-Suazo et al., 1999; Murray et al., 2006). In the present study, an association of high levels of IFN-γ and increased parasite load after 28 days was observed with Glucantime^®^, as well as with EE or FA, especially at the highest dose tested, providing a possible mechanism for parasite death *in vivo*. However, we confirmed that other cytokines may be involved in this process, highlighting the need for more comprehensive investigations of the cytokine profile during infection and treatment.

The susceptibility phenotype of *L. amazonensis* infection is clearly associated with elevated IL-4 levels and the Th2 response (Kopf et al., 1996). IL-4 reduces iNOS expression and increases disease progression due to increased survival and parasite growth in infected cells (Hurdayal et al., 2013). Furthermore, high levels of IL-10 also participate in this process, as the cytokine causes inhibition of macrophage activation and contributes to the growth of parasites in lesions (Kane and Mosser, 2001).

Our results demonstrated that decreased IL-10 expression in groups treated with Glucantime^®^, EE or FA were associated with decreased parasite load, while high levels of IL-10 expression were associated with increased parasite load in mice with simulated treatment, confirming the role of IL-10 in maintaining the infection and the immunomodulatory activity of EE and FA.

Cytokines measured in serum revealed an IL-10 increase and a decrease in IFN-y in untreated *L. amazonensis* infected animals, which were not observed after 28 days of treatment with EE, FA or Glucantime^®^. Decreased IL-10 and increased IFN-y levels contributed to maintain a Th1 response in the treated groups. Furthermore, the increase in IFN-γ after 28 days of treatment with Glucantime^®^ and with the extracts contribute to the effectiveness of macrophages by inducing iNOS in the skin and decreasing the parasite load. In fact, both extract and FA treatment decreased IL-10 and increased IFN-y levels.

Together, these results support the immunomodulatory effects of *A. nitidum*. Studies demonstrate that immunochemotherapy is more effective than chemotherapy or immunotherapy alone (Joshi et al., 2014), and our results show that treatment with *A. nitidum* is an immunochemotherapy.

When the reduction of IL-10 is observed in the groups treated with *A. nitidum* and Glucantime^®^, there is a difference in the percentage of reduction. However, positive control needs to be parenterally injected, while EE can be orally administered, and this is very important in long-term treatment.

Furthermore, while our histopathological analysis of the skin suggested that both EE and FA helped control inflammatory infiltrates and initiate the remodeling process, it is important to consider that the lack of direct measurement of cytokines and inflammatory cells in the tissue of a mice future investigation including such direct analyzes would be crucial to a more complete understanding of the affected mechanisms involved in *Leishmania* infection and response to EE and FA treatment (Murray et al., 2006).

The tissue repair process is critically important for the rapid cure of cutaneous leishmaniasis (Sakthianandeswaren et al., 2005). Thus, the control of inflammatory process and tissue remodeling mediated by EE or FA should be associated with a reduction in IL-10 and an increase in IFN-γ (Corware et al., 2011) and TGF-β (Castellucci et al., 2012). In uninfected wounds, high levels of IL-10 decrease inflammation, normal collagen deposition, and restore the normal dermal architecture (Peranteau et al., 2008), while TGF-β and IFN-γ induce recruitment of immune cells and promotes matrix protein synthesis, while decreases matrix protein degradation, leading to fibrotic tissue formation (Goldberg et al., 2007).

To validate our docking methodology, we conducted a redocking of the crystallographic ligand into the binding pocket of the Trypanothione reductase (TR) protein from *Leishmania amazonensis*, achieving an RMSD value of 1.3 Å. This validation confirms that our docking protocol accurately replicates known interactions (Forli et al., 2016; Silva et al., 2023). The binding affinities of yohimbine, corinanteol, and dihydrocorinanteol were favorable, with Moldock scores of −65.37, −76.69, and −74.08, respectively. The interaction of these compounds with critical catalytic residues—Cys52, Cys57, His461’, and Glu466’—highlights their potential as effective TR inhibitors, as these residues are essential for the protein’s redox mechanism (Baiocco et al., 2009).

Furthermore, the molecular dynamics (MD) simulations demonstrated the structural stability of the TR-ligand complexes over a 100 ns trajectory, with minimal conformational changes observed in the ligands. The binding affinities were confirmed through the MM-GBSA method, showing interaction energies of −21.72, −20.21, and −22.15 kcal/mol. Given the importance of TR in *Leishmania* metabolism, our findings underscore its potential as a drug target for antileishmania therapies. Previous studies have indicated that targeting TR can lead to effective therapeutic interventions against leishmaniasis (Battista et al., 2020; Santiago-Silva et al., 2023). By integrating our *in silico* results with existing literature, we emphasize the therapeutic relevance of TR inhibitors in combating leishmaniasis and encourage further investigation into these promising compounds.

The mechanism of action of alkaloids on parasites seems to involve binding with the DNA topoisomerase complex (Brandão et al., 2010; Brígido et al., 2020) and inhibition of oligopeptidase B (Cahlíková et al., 2013; 2015), a cytosolic protein belonging to the prolyl oligopeptidase family of serine proteases, responsible for the regulation of the levels of enolase on the cell surface of *Leishmania* parasites, which contributes to parasite virulence (Barrett and Rawlings, 1995; Rawlings and Barrett, 1999). However, the immunomodulator mechanism seems to involve the elevation of IFN-γ and IL-10, described for the first time in the present study.

Our study suggests that *A. nitidum* may have potential in reducing parasite load and lesion size in BALB/c mice infected with *L. amazonensis*. However, the effectiveness of the treatment has not yet been fully proven by the results presented. Furthermore, we observed immunomodulatory effects on cytokine expressions, which suggests a possible mechanism of action. These results highlight the importance of further investigations to fully understand the therapeutic potential of *A. nitidum* in cutaneous leishmaniasis.

## Data Availability

The raw data supporting the conclusions of this article will be made available by the authors, without undue reservation.
